# Estradiol induces apoptosis via activation of miRNA-23a and p53: implication for gender difference in liver cancer development

**DOI:** 10.18632/oncotarget.5472

**Published:** 2015-09-30

**Authors:** Fung-Yu Huang, Danny Ka-Ho Wong, Wai-Kay Seto, Ching-Lung Lai, Man-Fung Yuen

**Affiliations:** ^1^ Department of Medicine, The University of Hong Kong, Queen Mary Hospital, Hong Kong SAR; ^2^ State Key Laboratory for Liver Research, The University of Hong Kong, Hong Kong SAR

**Keywords:** estrogen, microRNA, gender difference, apoptosis, hepatocellular carcinoma

## Abstract

Estrogen (E2) has been suggested to have a protective role in attenuating hepatocellular carcinoma (HCC) development. miRNAs have great potential as biomarkers and therapeutic agents owing to their ability to control gene expression. However, little is known about the mechanism underlying the protective role of E2 in hepatocarcinogenesis and the effects of E2 on apoptotic miRNAs expression. Using miRNA PCR array, we found more than 2-fold alteration was observed in 25 upregulated and 10 downregulated apoptotic miRNAs in E2-treated cells. Among these miRNAs, we found expression of miR-23a was related to p53 functional status in the male-derived liver cell-lines. We demonstrated that E2 via ERα transcriptionally activated miR-23a and p53 expression, and thus enhanced p53 activation of miR-23a expression. Moreover, miR-23a expression correlated inversely with the expression of target gene X-linked inhibitor of apoptosis protein (XIAP), but positively with the caspase-3/7 activity. Decreasing of XIAP might contribute to caspase-3 activity and cell apoptosis. Taken together, our findings reveal a novel E2-signaling mechanism in regulating miRNAs expression for controlling apoptosis in liver cells. Delineating the role of E2 in regulating the activation of p53 and miR-23a, expression in HCC is crucial to the understanding of the sex difference observed in HCC.

## INTRODUCTION

Hepatocellular carcinoma (HCC) is the fifth most common malignant cancer and the third leading cause of cancer-related death in the world. The development of HCC is considered to be the most serious result of chronic liver diseases, including viral hepatitis B and C, cirrhosis due to other causes and alcoholic liver diseases [1]. Although the incidence of HCC worldwide is increasing [2], our understanding of the underlying molecular pathogenesis pathways in HCC is still limited.

Epidemiological data indicate that regardless of etiology, the incidence of HCC is higher in males than in females, with a male to female ratio ranging between 3–5 to 1 [3]. This sex difference is also found in the development of cirrhosis. This favourable outcome in female patients is attenuated after menopause [4]. Both epidemiologic evidence and results from animal studies have shown that estrogen (E2) may have a tumor protective role in female [4–6]. However, the mechanisms governing these phenomena are largely unknown.

MicroRNAs (miRNAs) are a family of small noncoding RNA molecules that regulate target genes expression at the post-transcriptional level. They are involved in stem cell self-renewal, cellular development, proliferation, and apoptosis [7]. Growing evidence indicates that miRNA is differentially expressed during development of many types of cancer, including liver cancer [8–10]. These findings suggest that miRNAs may play an important role in carcinogenesis as a novel class of oncogenes or tumor-suppressor genes. Thus, miRNAs have been considered as potential targets for cancer therapy as well as diagnostic tools.

The homeostatic balance between cell proliferation and cell death rate is critical for maintaining normal physiological processes. Aberrant regulation of apoptotic cell death mechanisms is one of the hallmarks of cancer development and progression, and many cancer cells exhibit significant resistance to apoptosis signaling [11]. Increasing number of miRNAs have been implicated in regulating cell death [12]. They either function as pro-apoptotic or anti-apoptotic miRNAs which regulate target anti-apoptotic or pro-apoptotic mRNAs expression respectively, and thus regulate cell apoptosis [13, 14]. Deregulation of these apoptotic-miRNAs might contribute to the development of disease, including cancers. Recent investigations have reported that miRNAs are involved in E2 signaling pathway [15, 16]. We therefore speculate that some of these miRNAs may contribute to the unique gender disparity observed in HCC. Particularly, it will be interesting to determine if E2 plays a role in regulating specific apoptotic miRNA(s) expression, thus inhibiting HCC development.

The aims of this study were to 1) evaluate the effects of E2 on apoptotic miRNAs expression; 2) explore the possible molecular mechanisms underlying the differential expression of miR-23a observed in HCC cells; and 3) identify whether E2-regulated miRNA-23a subsequently control target genes expression and cell apoptosis in HCC.

## RESULTS

### Identification of estrogen-regulated apoptotic miRNAs

**Figure 1:**
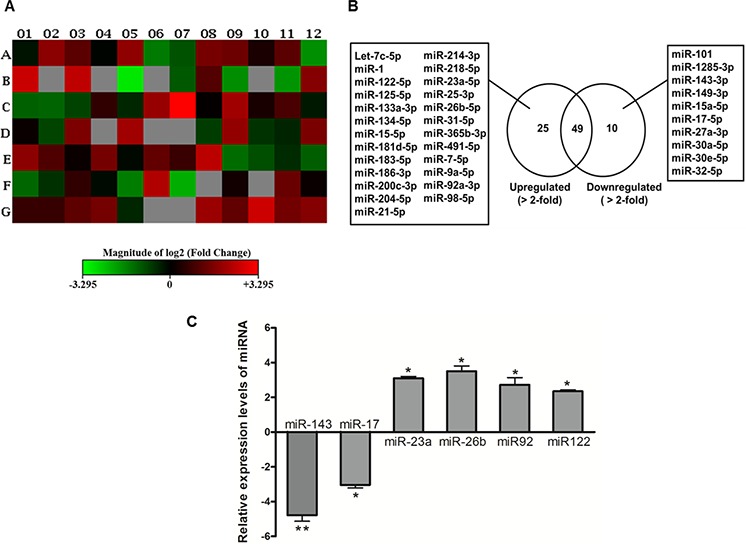
Deregulation of apoptotic miRNA expression in SNU-387 liver cancer cells following 10^−8^M estrogen (E2) treatment for 24 hours **A.** Heat map generated from PCR array data reflecting miRNAs expression values in E2-treated cells versus untreated controls. This graph represents log2 fold-regulation data from two sample groups on a 96-well plate layout. Red indicates up-regulation; green indicates down-regulation; black indicates no change; and grey indicates not determined (the recommended lower limit of detection when both samples with Ct ≥ 35). The bar code on the bottom represents the color scale of the log2 values. **B.** List of the miRNAs that were upregulated (>2.0 fold) and downregulated (>2.0 fold) in E2-treated cells as determined by miRNA PCR array analysis. **C.** Confirmation of six aberrantly expressed miRNAs using quantitative real-time PCR. Cells were starved overnight in serum-free medium before treatment with 10^−8^M E2 for 24 hours. The expression of miRNAs was normalized with internal control U6 SnRNA and compared with vehicle-treated control cells calculated using ΔΔ*Ct* method. Data are expressed as means ± SEM from three individual experiments. **p < 0.05 and *p < 0.01.

We profiled the miRNA expression in response to E2 treatment in SNU-387 cells. Among the 84 apoptotic miRNAs analyzed (Figure 1A), 25 miRNAs were upregulated (>2.0 fold), whereas 10 miRNAs were downregulated (>2.0 fold) compared with the untreated control cells (Figure 1B and Supplementary Table S2). Based on their levels of distinct differential expression, as well as their relevance to cancer development as revealed by literature review [13, 17–21], we selected 6 miRNAs for further validation. Microarray results were confirmed by evaluating the expression of miRNAs using real-time PCR. We found miR-23a, miR-26b, miR-92 and miR-122 were significantly upregulated (all with *p* < 0.05), whereas miR-143 and miR-17 were significantly downregulated when compared with untreated control cells (*p* < 0.01 for miR-143; *p* < 0.05 for miR-17) (Figure 1C). This indicated that the real-time PCR validation results were consistent with the microarray measurements.

### In silico analysis of miRNA target genes

We speculated that these E2-regulated miRNAs might play a role in the regulation of apoptosis in HCC. Therefore, we performed an in silico search for target genes of these miRNAs, with special focus on apoptosis-related genes. E2 acts through estrogen receptors (ERs). miR-23a processes four ERα-biding sites in its regulatory region [22], and is implicated in the regulation of several important apoptosis-related genes (Supplementary Table S3). Thus, miR-23a was chosen to be a candidate to investigate apoptotic miRNA involvement in E2 signaling and selected for further analysis.

### Endogenous expression of miR-23a in liver cancer cell-lines

**Figure 2:**
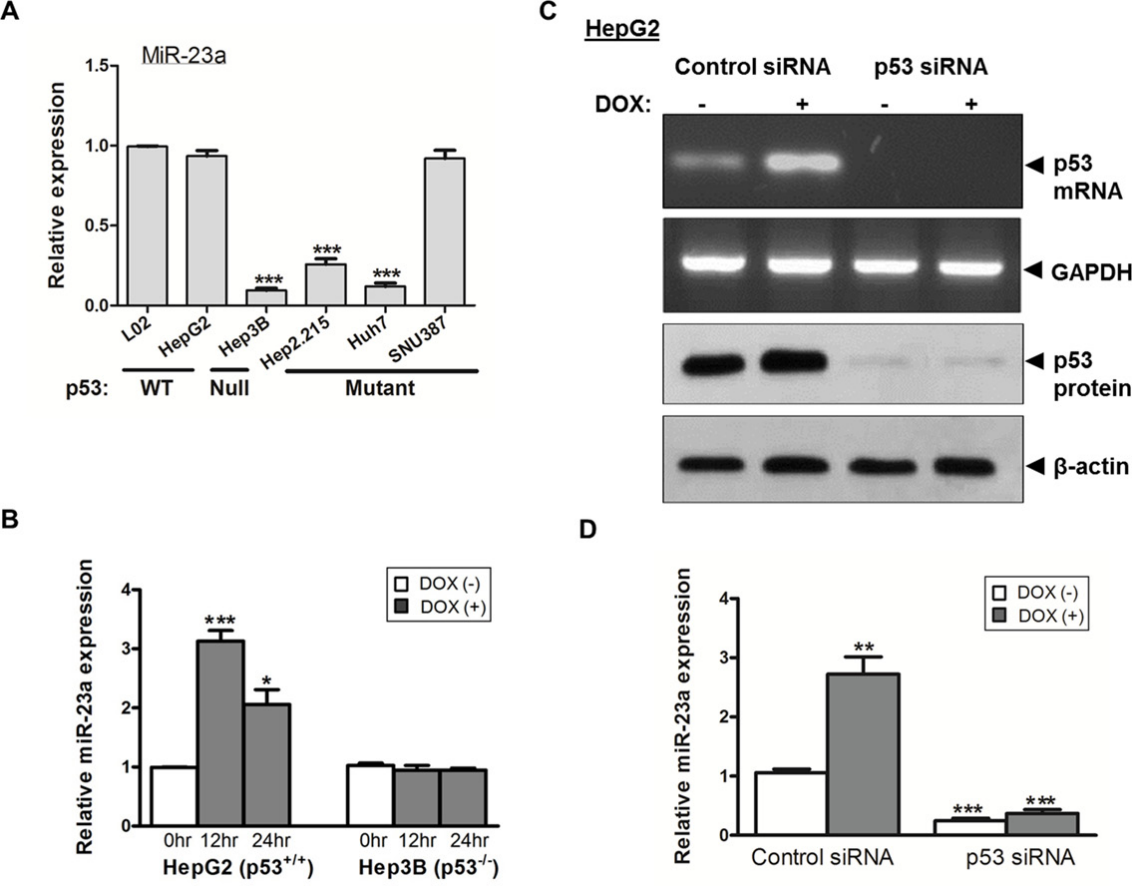
Expression of the tumor suppressor p53 and miR-23a in liver cancer cells **A.** Differential expression of miR-23a in relation to the p53 functional status in liver cancer cell-lines. **B.** Effect of doxorubicin (DOX) on the expression of miR-23a in p53^+/+^ HepG2 and p53^−/−^ Hep3B cells. **C.** Transient knockdown of p53 by p53-siRNA suppressed p53 mRNA and protein expression. **D.** Effect of p53 knockdown and DOX treatment on miR-23a expression in HepG2 cells (***p < 0.0001, **p < 0.001 and *p < 0.01).

We measured the expression of miR-23a in 6 liver cell-lines. As shown in Figure 2A, miR-23a expression in the female-derived cell line SNU-387 was significantly higher than that in three of the male-derived cell-lines Hep3B, Huh7 and HepG2.2.15 (*p* < 0.001). However, miR-23a expression in the other 2 male liver cell-lines, L02 and HepG2, was comparable to SNU-387. Notably, among the 5 male liver cell-lines, the expression levels of miR-23a were related to the mutational status of the tumor suppressor gene p53 in the cells (Figure 2A). When compared with cell-lines with wild-type p53 (L02 and HepG2), cell-lines with null/mutated p53 (Hep3B [p53-null], Huh7 [p53-mutated] and HepG2.2.15 [p53-LOH]) had significantly lower expressions of miR-23a (all *p* < 0.001). Although the female cell-line SNU-387 has a p53-heterozygotic genotype [23, 24], its endogenous miR-23a expression level was comparable to that in L02 and HepG2 cells with wild-type p53 (Figure 2A).

### P53-induced the expression of miR-23a

To confirm whether the difference in miR-23a expression was related to p53 functional status, we altered the p53 activity in HepG2 (p53^+/+^) and Hep3B (p53^−/−^) and measured their subsequent effects on miR-23a expression. As shown in Figure 2B, following treatment of cells with doxorubicin (DOX), a potent p53 inducer, miR-23a expression levels was significantly increased in HepG2 cells when compared with non-treated control cells (*p* < 0.0001 at 12 hr; *p* < 0.01 at 24 hr). In contrast, DOX treatment did not induce the expression of miR-23a in Hep3B cells at all the time points (Figure 2B). This observation was further confirmed by siRNA-mediated p53 knockdown in HepG2 cells (Figure 2C). As shown in Figure 2D, in HepG2 cells transfected with control siRNA, DOX treatment significantly increased the expression of miR-23a (*p* < 0.001) when compared with cells without DOX treatment. However, in HepG2 cells with siRNA-mediated p53 knockdown, the expression of miR-23a was significantly lower when compared with cells transfected with control siRNA, regardless of the presence or absence of DOX, (both *p* < 0.001) (Figure 2D).

**Figure 3:**
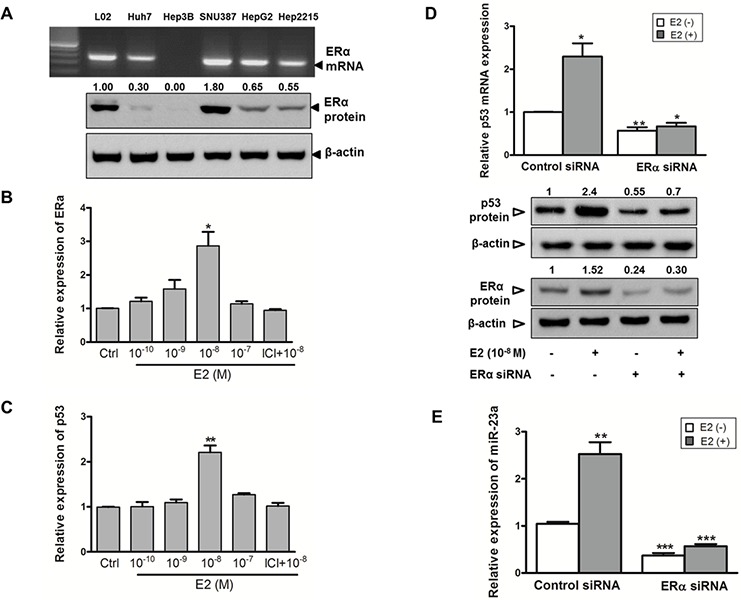
Estrogen induces p53 and miR-23a expression in SNU-387 cells **A.** Endogenous mRNA and protein expression of estrogen receptor ERα was assessed in liver cell-lines. **B.** and **C.** ERα and p53 mRNA expression in SNU-387 cells with E2 treatment (10^−10^-10^−7^M) or with pretreatment of 1 μM ICI-182,780 (ICI) for 24 hours were analyzed by quantitative RT-PCR. **D.** Relative mRNA and protein expression of p53 and protein expression of ERα with or without knockdown of ERα by ERα siRNA was measured in SNU-387 cells. **E.** Quantitative RT-PCR analysis of the effects of E2 on miR-23a expression in SNU-387 cells with or without ERα knockdown. Data are expressed as means ± SEM from three individual experiments (***p < 0.0001, **p < 0.001 and *p < 0.01).

The above results indicated that, in the male-derived HepG2 cells, both expression and activation of p53 were crucial to augment miR-23a expression. However, in the female-derived SNU-387 cells, despite its p53-heterozygotic genotype, its miR-23a expression levels were comparable with other cell-lines with functional p53 (Figure 2A). As miR-23a expression in SNU-387 was upregulated after E2 treatment (Figure 1 and Supplementary Table S2), we studied whether E2 affected p53 expression. As shown in Figure 3C, the expression of p53 in SNU-387 was significantly increased when treated with 10^−8^M E2 (*p* < 0.001; compared with untreated controls), indicating that E2 may involve in p53-mediated regulation of miR-23a expression.

### Estrogen increases p53 and miR-23a expression via estrogen receptor-α

Next, we studied whether the effects of E2 on miR-23a and p53 expression was mediated via the ERα receptor. As shown in Figure 3A, SNU-387 cells had a higher expression of ERα than other male-derived cell-lines. When SNU-387 cells were treated with E2, an increased in ERα mRNA expression was observed, with a maximum increase of nearly 3-fold at 10^−8^M E2 (*p* < 0.01) (Figure 3B). In Hep3B cells, in which no expression of ERα was detected (Figure 3A), treatment of E2 did not induce the expression of both ERα and p53 (data not shown). This might account for the low expression of miR-23a in Hep3B cells (Figure 2A).

To further confirm that E2 regulates the expression of p53 through ERα, we pretreated SNU-387 with the ERα antagonist ICI 182,780 and found that it completely blocked the induction of ERα and p53 expression by E2 (Figure 3B and 3C). In addition, transient knockdown of ERα was achieved by siRNA-ERα in SNU-387 cells with or without E2 treatment that led to a decrease in p53 mRNA (*p* < 0.01 and *p* < 0.001 respectively) and protein expression levels (Figure 3D). Consistent with the above observations, knockdown of ERα decreased the expression of miR-23a in SNU-387 cells with or without E2 treatment (both *p* < 0.0001) (Figure 3E). These results indicated that upon ligand E2 binding, ERα activates both p53 and miR-23a expression at the transcriptional level.

### The effects of miR-23a on expression of anti-apoptotic protein XIAP

**Figure 4:**
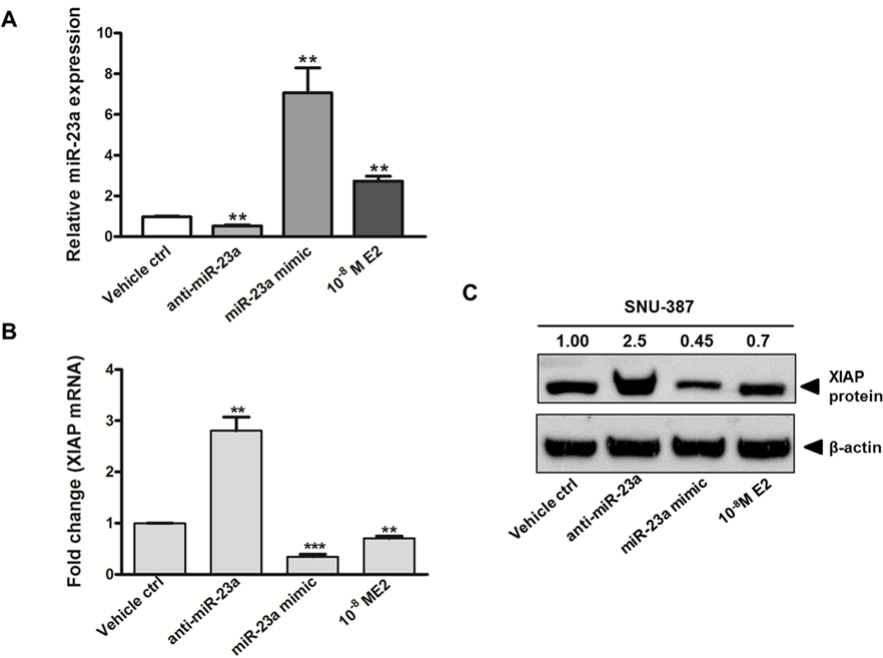
Gain of function and loss of function analysis on the expression of miR-23a and its effect on X-linked inhibitor of apoptosis protein (XIAP) expression in SNU-387 cells Quantitative analysis on **A.** miR-23a expression; **B.** XIAP mRNA expression; and **C.** XIAP protein expression in SNU-387 cells transfected with miR-23a mimic (20 nM) or anti-miR-23a (20 nM), or with E2 (10^−8^M) treatment. Cells were collected for analysis at day 2 after treatment or transfection. Data represent means ± SEM from three independent experiments (***p < 0.0001, **p < 0.001 and *p < 0.01).

We also studied the expression of other target genes of miR-23a. Among the potential targets of miR-23a (Supplementary Table S3), the X-linked inhibitor of apoptosis protein (XIAP) was chosen for further analysis. XIAP is the most potent anti-apoptotic protein and has been demonstrated to be overexpressed in HCC and counteract apoptosis [25–27]. Furthermore, in silico analysis predicted that there were five potential miR-23a-binding sites in the 3′-UTR of XIAP (Supplementary Figure S1). The interaction between miR-23a and XIAP in SNU-387 cells was confirmed by gain-of-function and loss-of-function approaches. Transfection of cells with anti-miR-23a, which decreased the level of endogenous miR-23a (Figure 4A), led to a significant increase in the expression of the target XIAP at both mRNA and protein levels (*p* < 0.001) (Figure 4B and 4C). Similarly, transfection of cells with miR-23a mimic, which significantly increased the level of miR-23a (*p* < 0.001) (Figure 4A), resulted in the suppression of target XIAP at both mRNA and protein levels (*p* < 0.0001) (Figure 4B and 4C). When cells were treated with E2, miR-23a expression was augmented and XIAP expression was decreased (both *p* < 0.001) (Figure 4B and 4C). These findings suggest that XIAP expression is down-regulated by miR-23a.

### E2, miR-23a, and cell apoptosis

**Figure 5:**
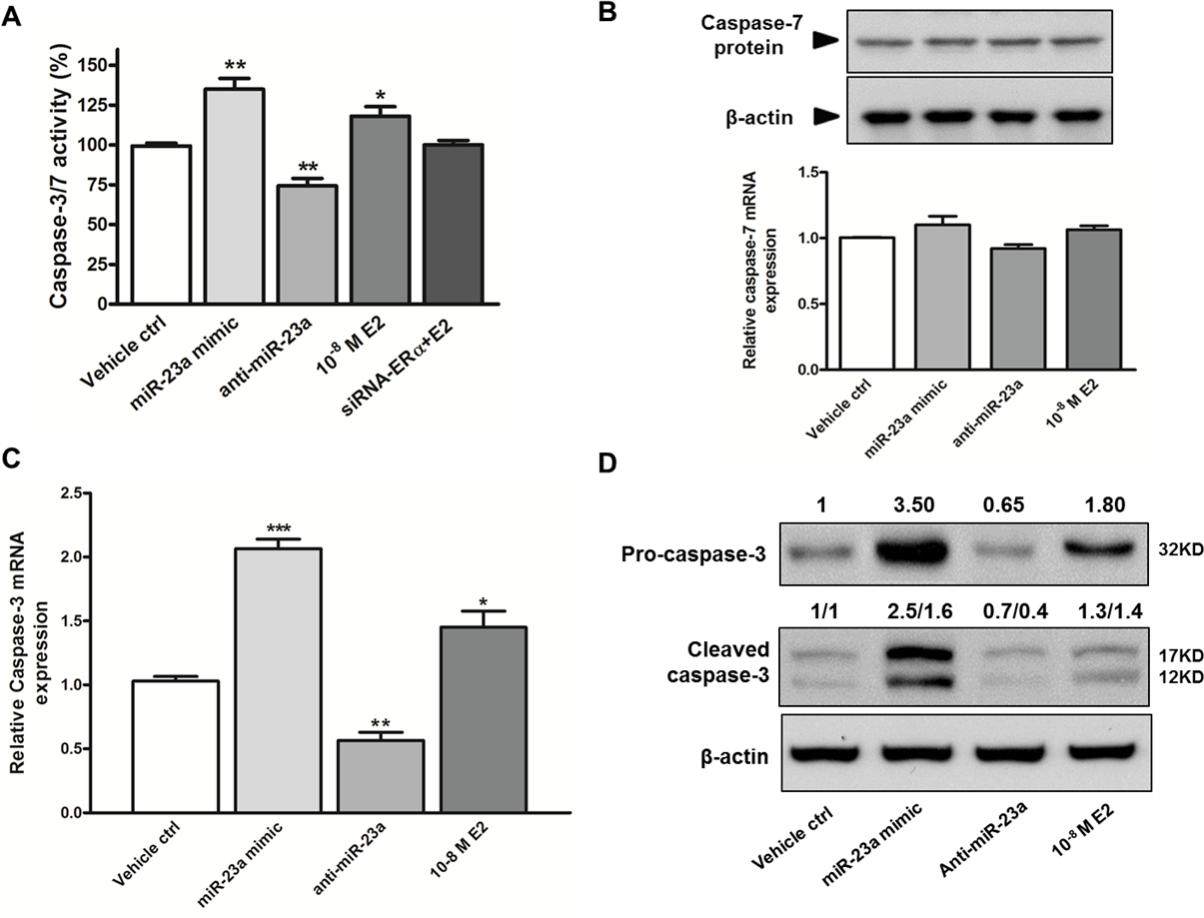
Effects of miR-23a on caspase-3/7 activity and apoptosis in SNU-387 cells **A.** Caspase 3/7 activity were analyzed in SNU-387 cells transfected with miR-23a mimic or anti-miR-23a, or with E2 treatment +/− ERα knockdown (**p < 0.005 and *p < 0.05) for 24 hours. Similarly, effect of miR-23a mimic, anti-miR-23a, and E2 treatment on **B.** caspase-7 mRNA and protein expression; **C.** caspase-3 mRNA expression; and **D.** pro-caspase-3 (32KD) and active caspase-3 (17KD) protein levels were analyzed 24 hours after transfection or treatment in SNU-387 cells. Data are mean values ± SEM from three independent experiments (***p < 0.0001, **p < 0.001 and *p < 0.01).

XIAP inhibits cell death by binding to apoptotic executioners, caspase-3/7. Therefore, we assessed whether miR-23a regulates the activity of caspase-3/7 in SNU-387 cells. As shown in Figure 5A, the activity of caspase-3/7 was elevated by 18% in E2 treated cells when compared with control cells (*p* < 0.05). This induction of caspase-3/7 activity was abolished by transient knockdown of ERα. To further prove E2 increases caspase-3/7 activity via miR-23a, we transfected miR-23a mimic or anti-miR-23a into SNU-387 and found that miR-23a mimic increased caspase-3/7 activity by 35% and anti-miR-23a suppressed caspase-3/7 activity by 26% (both *p* < 0.005; Figure 5A).

In addition to caspase-3/7 activity, we measured the expression of capsase-3/7. Caspase-7 is also a predicted target of miR-23a (Supplementary Table S3). Neither E2 nor miR-23a had any effect on caspase-7 mRNA and protein expression (Figure 5B). This suggested that caspase-7 expression was not regulated by miR-23a in liver cells, and the induction in caspase-3/7 activity was likely due to activation of caspase-3. This was confirmed by the increase in caspase-3 expression in cells treated with E2 (*p* < 0.0001) and miR-23a mimic (*p* < 0.01) and the decreased caspase-3 expression in anti-miR-23a transfected cells (*p* < 0.001; Figure 5C). In concordance with the above findings, E2 and miR-23a mimic increased pro-caspase-3 (32kD) and active caspase-3 (17kD) protein expressions, whereas anti-miR-23a suppressed both forms of caspase-3 protein expressions (Figure 5D). These results indicate that miR-23a decreased XIAP expression, which subsequently induced caspase-3 expression and activity.

**Figure 6:**
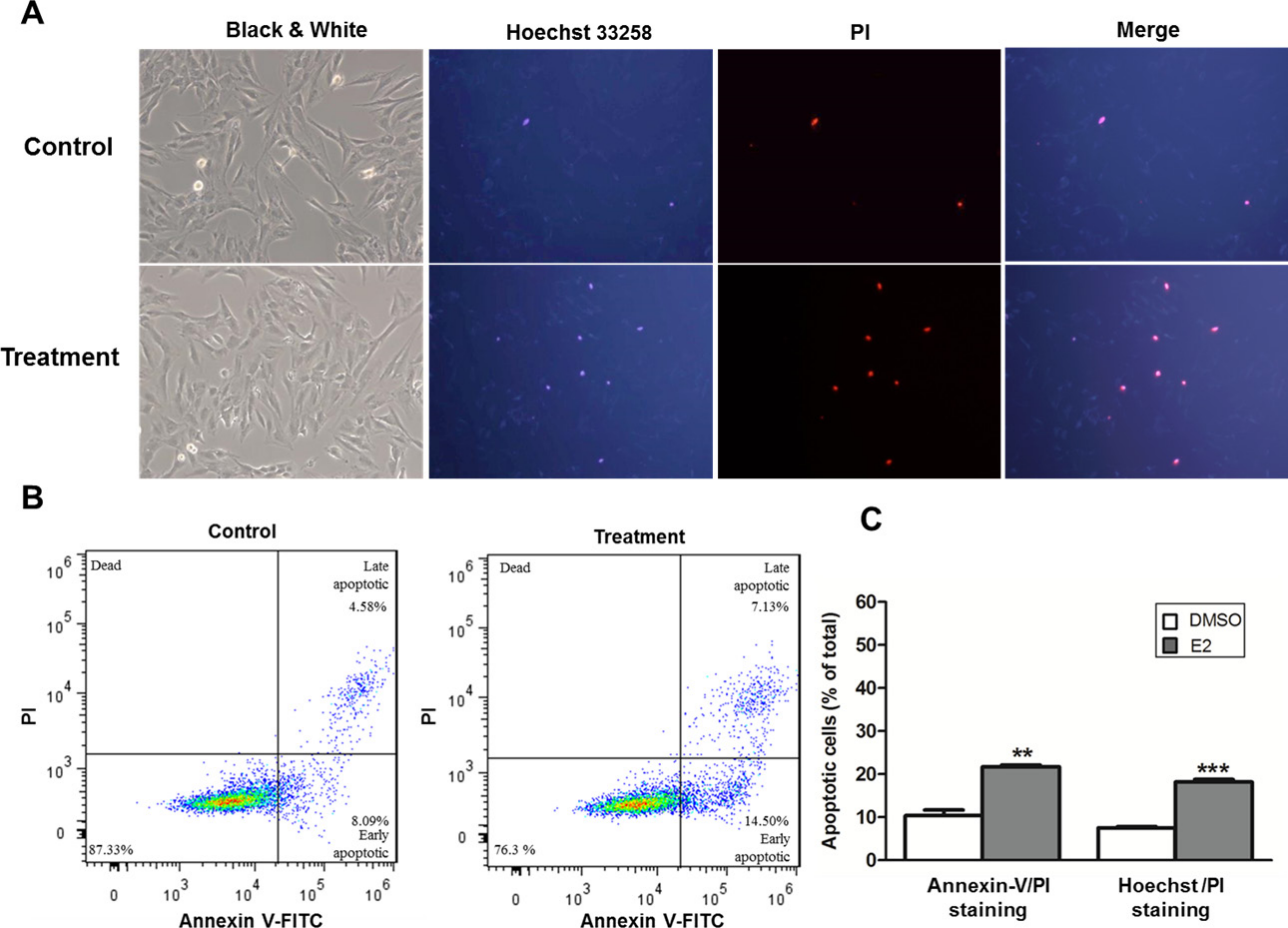
Estrogen induced apoptosis in SNU-387 cells The cells were collected for analysis after treatment with 10^−8^M E2 for 24 hours. **A.** Representative fluorescence images obtained after Hoechst 33258 and propidium iodide (PI) double staining. The nuclei of apoptotic cells have highly condensed chromatin that is stained by Hoechst 33258 and PI, indicating by bright blue and red spherical nucleus respectively. At least 200 cells in 6 randomly selected areas were counted in treatment and control cells **B.** Flow cytometry analysis of apoptosis by double staining with Annexin-V FITC/PI. Numbers indicate the percentage of cells in each quadrant **C.** The percentage of apoptotic cells (A and B) compared with control cells treated with vehicle (DMSO), respectively was counted from three independent experiments. All data were expressed as means ± SEM (**p < 0.001 and ***p < 0.0001).

Finally, to extend the above findings on activation of caspase 3 and cell death, we assessed the apoptotic population in E2-treated SNU-387 cells by Hoechst/PI double staining and flow cytometry analysis. As shown in Figure 6A, the number of cells underwent apoptotic cell death was higher in E2 treated group than controls, as characterized by condensation of chromatin with Hoechst/PI double staining. This was consistent with the data obtained from flow cytometry analysis. E2 treatment increased the percentage of early (Annexin-V FITC positive) and late (Annexin-V FITC and PI positive) apoptotic cells with relative to control cells without treatment (Figure 6B). Results of both analyses showed significant increase in the number of apoptotic cells in SNU-387 with E2 treatment compared with control cells (*p* < 0.0001) (Figure 6C). The apoptotic cell death with E2 treatment, which increased with time confirming the apoptotic effect of E2 in SNU-387 cells were showed in Supplementary Figure S3. Taken together, these findings suggest a novel relationship between E2 treatment, miR-23a activation and the commitment of the cells to apoptotic pathway.

## DISCUSSION

Deregulation of several miRNAs have been reported to be involved in controlling apoptosis and carcinogenesis [12]. E2 has been implicated in gender disparity and attenuated HCC development [4, 28]. However, attempts to determine miRNAs as posttranscriptional regulators in E2 signaling in liver cells are scarce. E2 regulates gene expression by binding to its receptors (ERs) [29]. The activated ERs then translocate into the nucleus, bind to DNA, and regulate target gene transcription. As miRNAs are themselves genes, they too can also be regulated in this manner. Indeed, recent studies have demonstrated that ERs bind to regulatory regions upstream of miRNAs and stimulate their transcription [30].

Consistent with previous reports in other types of cancer [15, 16, 31, 32], we found that E2 treatment regulated apoptotic miRNAs expression in HCC. Our miRNA PCR array analysis revealed a >2−fold upregulation in 25 miRNAs and a >2−fold downregulation in 10 miRNAs. A 2-fold difference in one single miRNA has been considered to be significant in decreasing the expression of multiple target genes and proteins resulting in remarkable physiological effects [33, 34]. Notably, we found E2 treatment upregulated the tumor suppressive miRNAs, miR-26a, miR-92 and miR-122, and suppressed the oncomirs miR-143 and miR-17. Expression of these tumor suppressive miRNAs and oncomirs have been reported to be down-regulated and elevated in HCC, respectively [20, 35–39]. The role of miR-23a has been reported with varying conclusions. Some early studies showed that miR-23a promotes cell proliferation and anti-apoptosis [40, 41]. More recent studies demonstrated that miR-23a could induce caspase-dependent and -independent apoptosis in human embryonic kidney cells [42], and granulosa cells [43]. The different effects of miR-23a may be related to the fact that the physiological effects of miRNAs are cell-type dependent and disease specific [44]. In this study, we found E2 treatment induced the expression of miR-23a. This is consistent with previous reports indicating that miR-23a is an E2-responsive miRNA [15, 45].

To our knowledge, the present study is the first to show that miR-23a expression was related to functional status of p53 in HCC cell-lines. We found that expression of miR-23a in male liver cell-lines was dependent on a functional p53. Unexpectedly, in the female cell-line SNU-387, expression of miR-23a was not completely p53-dependent. Thus, other transcriptional mechanisms in addition to p53 signaling might contribute to miR-23a expression. We therefore speculated that there was a link between E2 and p53 on the activation of miR-23a expression. We found that E2 treatment induced the expression of both p53 and ERα in an E2-dependent manner (Figure 3B and 3C), and transiently knockdown of ERα abolished the induction of both p53 and miR-23a expression (Figure 3D and 3E). This observation suggested that upon E2 treatment, ERα might induce miR-23a expression either directly by binding to miR-23a regulatory regions or synergistically by binding to regulatory region of p53 to activate its expression, and thus increase the expression of miR-23a. In SUN-387 cells, even one allele of p53 is deficient, the effect of E2 seemed to overcome the deficit in p53 activity in induction of miR-23a expression. Our findings indicate that E2 treatment has a strong influence on miR-23a expression, which in turn affects apoptosis.

Apoptosis serves as a natural constraint to cancer formation and development. Overexpression of XIAP that counteracts apoptosis has been demonstrated in HCC [25, 27]. In this study, we observed a clear inverse relationship between the changes of miR-23a levels and the expression of its corresponding target gene, XIAP. This E2-induced reduction of XIAP expression was mediated by ERα and was associated with an increased caspase-3 mRNA and protein expression and enhanced caspase 3/7 activity. These findings suggest that E2-activated ERα exerts a post-transcriptional control on its target gene XIAP, via miR-23a, and decrease in XIAP expression may contribute to the activation of caspase-3 and induction of cell death.

Genome-wide decreases in the miRNAs expression are common in malignancy and this in turn contributes to aberrant mRNA expression patterns observed in primary tumors [10]. p53 has been shown to regulate expression of miRNAs involved in tumor suppression and cellular senescence [46, 47]. Loss of p53 function can sensitize cells to DNA damage due to impaired DNA repair. As a result, damaged cells are less likely to undergo apoptosis which may lead to malignant transformation [48]. In this study, we found E2 treatment increased p53 expression and subsequently activated miR-23a, thereby suppressing XIAP expression and causing cell-death. Therefore, it is reasonable to postulate that E2 exerts a tumor suppressor role in HCC by activation of p53 expression and apoptosis. This may also explain the sex disparity observed in HCC development. In fact, miR-23a mediated XIAP expression has been suggested to have a protective effect and contributes to the sex difference in brain injury in mice [49]. Furthermore, suppression of miR-23a inhibited the activation of p53 target genes and cell death in HCC cells [50]. Taking these findings together, we propose a possible model for the effect of E2 on cellular apoptosis. Upon E2 treatment, ERα expression is activated, which in turns increases the expression of miR-23a either directly or through the synergistic effect of increased p53 expression. The increased miR-23a expression results in a decreased XIAP expression, which subsequently enhances caspase-3/7 activity through an increase in caspase 3 expression. As a result of the induction of both p53 and caspase-3/7, cellular apoptosis is enhanced.

In conclusion, this study identified a novel relationship between E2, p53 and miR-23a in liver cancer cells. Their interactions suggested a possible mechanism by which E2 induced cellular apoptosis and hence conferred a protective role against HCC. Further delineation of the E2-signaling mechanism in regulating the activation of p53 and miR-23a expression in HCC will be crucial to the understanding of the sex difference observed in this disease entity, and may provide a new avenue for the development of more efficient therapeutic strategies and treatment.

## MATERIALS AND METHODS

### Cell lines and treatment

The five human liver cancer cell lines (SNU-387, Huh7, HepG2, HepG2.2.15 and Hep3B) were obtained from the ATCC. The L02 cell line derived from human normal liver was obtained from the Shanghai Institutes for Biological Sciences, and Chinese Academy of Sciences. Only SNU-387, HepG2.2.15 and Hep3B are HBV positive cell lines. Cells were cultured in RPMI-1640 medium without phenol red, supplemented with 10% charcoal-stripped fetal bovine serum (Invitrogen, Carlsbad, CA) in a humidified incubator with 5% CO_2_ at 37°C. SNU-387 was a female-derived liver cancer cell-line, and the others were male-derived cell-lines. Before treatment, cells were starved overnight in serum-free medium. Unless otherwise stated, cells were generally treated with 17β-estradiol (E2) (10^−8^M) or doxorubicin hydrochloride (DOX) (0.5 μM) (Tocris Bioscience, Ellisville, MO) for 24 hours.

### Transient miRNA and siRNA transfection

Transfection was performed with Lipofectamine 2000 reagent (Invitrogen) following the manufacturer's protocol. Briefly, 3 × 10^5^ cells were seeded in plates one day before transfection to ensure suitable cell confluency on the day of transfection. Oligonucleotides (miR-23a mimic and anti-miR-23a) (Ambion, Austin, TX) were used at a final concentration of 20nM in antibiotic-free Opti-MEM medium (Invitrogen). siRNAs (against ERα and p53) (Qiagen) were used at a final concentration of 50nM. siRNAs with non-specific sequences were used as controls. Cells were harvested at day 2 post-transfection.

### RNA isolation

Total RNA was extracted from cells using TRIzol reagent (Invitrogen), according to the manufacturer's instructions. DNA and RNA concentrations and integrity were determined using the NanoDrop 2000 Spectrophotometer (Thermo Fisher Scientific, MA).

### miRNA PCR array analyses

Two micrograms of total RNA isolated from E2-treated (10^−8^M for 24 hours) or vehicle-treated SNU-387 cells were reverse-transcribed using the miScript II RT kit (Qiagen), following the manufacturer's protocol. cDNA was assayed via an optimized, real-time PCR reaction for the expression of 84 apoptosis-related miRNAs using the human apoptosis miScript miRNA PCR array (Qiagen). Data analysis was performed with the online data analysis tool available at http://pcrdataanalysis.sabiosciences.com/mirna, using the ΔΔ*C_T_* method of relative quantification. Target genes were predicted using in silico analysis by PicTar and TargetScan algorithms [51, 52].

### Real-time PCR for miRNA and target genes expression

For miRNA analysis, mature miRNA was extracted from cell lines with or without drug stimulation or transfection (as described above). miRNAs were reverse-transcribed using the TaqMan MicroRNA reverse transcription kit (Applied Biosystems, Foster City, CA). Real-time PCR was performed using Taqman miRNA assays (assay ID: 000399, 000466, 000393, 000407, 000430, 002245) (Applied Biosystems), according to the manufacturer's instructions. U6 snRNA was not responsive to E2 treatment and stable among all cell-lines thus was used as the endogenous control (Supplementary Figure S2). For target gene analysis, total RNA was reverse-transcribed into cDNA using the Superscript III First-strand synthesis kit (Invitrogen). qPCR and semi-quantitative PCR were performed using the SYBR Green PCR master mix (Applied Biosystems) and ThermoScript™ RT-PCR System (Invitrogen), respectively. GAPDH was used as the internal control. Mean Ct was determined from triplicate PCRs. The relative expression level was then calculated using the ΔΔ*C_T_* method. Primer sequences are listed in Supplementary Table S1.

### Caspase activity assay

Caspase 3/7 activity was analyzed using the Caspase-GLO 3/7 kit (Promega, Madison, WI) as recommended by manufacturer's instructions. Following E2 treatment, SNU-387 and Hep3B cells were transfected with miR-23a mimic or anti-miR-23a in a 96-well plate. After 24 hours, cells were incubated with medium supplemented with 100 μl caspase-3/7 reagent for 3 hours at room temperature. Then, luminescence from cleaved caspase substrate was measured using the luminometer (CLARIOstar, BMG Labtech, Germany). Resulting data were expressed as OD values and normalized to untransfected control cells. Experiments were performed in triplicates.

### Apoptosis detection by flow cytometry and Hoechst 33258/propidium iodide double staining

SNU-387 cells were treated with E2 (10^−8^M) for 24 hours. Cells were harvested (including attached and detached cells), washed and resuspended with PBS. For flow cytometry analysis, apoptotic cells were determined with an Annexin V-FITC Apoptosis Detection Kit (BD Pharmingen, SD, USA) according to the manufacturer's protocol. Apoptosis cells were analyzed by using FC500 flow cytometer (Beckman Coulter, Germany). The data were analyzed using Flowjo software (Tree Star, OR, USA). For Hoechst 33258 (Sigma) and propidium iodide (PI) (Sigma) double staining analysis, cells were incubated with Hoechst 33258 (10 μg/mL) and PI (10 μg/mL) for 15 minutes, and then fixed in 4% formaldehyde for 20 minutes. Imaging was detected under a fluorescence microscope (Olympus, Tokyo, Japan) at 340 and 620 nm, respectively.

### Western blot analysis

Cells were lysed in RIPA buffer with 1 mM phenylmethylsulfonyl fluoride (Roche). Protein concentration was determined by BCA Protein Assay Kit (Thermo Fisher Scientific, MA USA). 20–50 μg of protein was loaded on 10% SDS-polyacrylamide gels, transferred to a PVDF membrane, blocked with 5% milk, and then probed with relevant primary antibodies to XIAP, ER-α, p53 (Santa Cruz, CA), caspase-3/7 and β-actin (Cell Signaling, MA) overnight at 4°C. Protein expression was assessed by ECL detection system (GE Healthcare, NJ) and band intensities were quantified using the Image J software (NIH, Bethesda, MD).

### Statistical analysis

Continuous variables were expressed as mean ± standard error (SEM) and analyzed using the student's *t*-test. All statistical analysis was done using GraphPad Prism 5.0 (GraphPad Software, Inc. San Diego, CA). A *p* value of less than 0.05 was considered statistically significant.

## SUPPLEMENTARY TABLES AND FIGURES





## References

[1] Parikh S, Hyman D (2007). Hepatocellular cancer: a guide for the internist. The American journal of medicine.

[2] Llovet JM, Burroughs A, Bruix J (2003). Hepatocellular carcinoma. Lancet.

[3] Bosch FX, Ribes J, Diaz M, Cleries R (2004). Primary liver cancer: worldwide incidence and trends. Gastroenterology.

[4] Shimizu I (2003). Impact of oestrogens on the progression of liver disease. Liver international : official journal of the International Association for the Study of the Liver.

[5] Naugler WE, Sakurai T, Kim S, Maeda S, Kim K, Elsharkawy AM, Karin M (2007). Gender disparity in liver cancer due to sex differences in MyD88-dependent IL-6 production. Science.

[6] De Maria N, Manno M, Villa E (2002). Sex hormones and liver cancer. Molecular and cellular endocrinology.

[7] Umpierrez GE, Latif K, Stoever J, Cuervo R, Park L, Freire AX, E Kitabchi A (2004). Efficacy of subcutaneous insulin lispro versus continuous intravenous regular insulin for the treatment of patients with diabetic ketoacidosis. The American journal of medicine.

[8] Ventura A, Jacks T (2009). MicroRNAs and cancer: short RNAs go a long way. Cell.

[9] Esquela-Kerscher A, Slack FJ (2006). Oncomirs - microRNAs with a role in cancer. Nature reviews Cancer.

[10] Lu J, Getz G, Miska EA, Alvarez-Saavedra E, Lamb J, Peck D, Sweet-Cordero A, Ebert BL, Mak RH, Ferrando AA, Downing JR, Jacks T, Horvitz HR, Golub TR (2005). MicroRNA expression profiles classify human cancers. Nature.

[11] Hanahan D, Weinberg RA (2000). The hallmarks of cancer. Cell.

[12] Jovanovic M, Hengartner MO (2006). miRNAs and apoptosis: RNAs to die for. Oncogene.

[13] Cimmino A, Calin GA, Fabbri M, Iorio MV, Ferracin M, Shimizu M, Wojcik SE, Aqeilan RI, Zupo S, Dono M, Rassenti L, Alder H, Volinia S, Liu CG, Kipps TJ, Negrini M (2005). miR-15 and miR-16 induce apoptosis by targeting BCL2. Proceedings of the National Academy of Sciences of the United States of America.

[14] Selcuklu SD, Yakicier MC, Erson AE (2009). An investigation of microRNAs mapping to breast cancer related genomic gain and loss regions. Cancer genetics and cytogenetics.

[15] Ferraro L, Ravo M, Nassa G, Tarallo R, De Filippo MR, Giurato G, Cirillo F, Stellato C, Silvestro S, Cantarella C, Rizzo F, Cimino D, Friard O, Biglia N, De Bortoli M, Cicatiello L (2012). Effects of oestrogen on microRNA expression in hormone-responsive breast cancer cells. Hormones & cancer.

[16] Paris O, Ferraro L, Grober OM, Ravo M, De Filippo MR, Giurato G, Nassa G, Tarallo R, Cantarella C, Rizzo F, Di Benedetto A, Mottolese M, Benes V, Ambrosino C, Nola E, Weisz A (2012). Direct regulation of microRNA biogenesis and expression by estrogen receptor beta in hormone-responsive breast cancer. Oncogene.

[17] Selcuklu SD, Donoghue MT, Spillane C (2009). miR-21 as a key regulator of oncogenic processes. Biochemical Society transactions.

[18] Zhuang M, Shi Q, Zhang X, Ding Y, Shan L, Shan X, Qian J, Zhou X, Huang Z, Zhu W, Cheng W, Liu P, Shu Y (2014). Involvement of miR-143 in cisplatin resistance of gastric cancer cells via targeting IGF1R and BCL2. Tumour biology : the journal of the International Society for Oncodevelopmental Biology and Medicine.

[19] Liu P, Tang H, Chen B, He Z, Deng M, Wu M, Liu X, Yang L, Ye F, Xie X (2015). miR-26a suppresses tumour proliferation and metastasis by targeting metadherin in triple negative breast cancer. Cancer letters.

[20] Shigoka M, Tsuchida A, Matsudo T, Nagakawa Y, Saito H, Suzuki Y, Aoki T, Murakami Y, Toyoda H, Kumada T, Bartenschlager R, Kato N, Ikeda M, Takashina T, Tanaka M, Suzuki R (2010). Deregulation of miR-92a expression is implicated in hepatocellular carcinoma development. Pathology international.

[21] Bandiera S, Pfeffer S, Baumert TF, Zeisel MB (2015). miR-122 - A key factor and therapeutic target in liver disease. Journal of hepatology.

[22] Bhat-Nakshatri P, Wang G, Collins NR, Thomson MJ, Geistlinger TR, Carroll JS, Brown M, Hammond S, Srour EF, Liu Y, Nakshatri H (2009). Estradiol-regulated microRNAs control estradiol response in breast cancer cells. Nucleic acids research.

[23] Ku JL, Park JG (2005). Biology of SNU cell lines. Cancer research and treatment : official journal of Korean Cancer Association.

[24] Lee JH, Ku JL, Park YJ, Lee KU, Kim WH, Park JG (1999). Establishment and characterization of four human hepatocellular carcinoma cell lines containing hepatitis B virus DNA. World journal of gastroenterology : WJG.

[25] Shiraki K, Sugimoto K, Yamanaka Y, Yamaguchi Y, Saitou Y, Ito K, Yamamoto N, Yamanaka T, Fujikawa K, Murata K, Nakano T (2003). Overexpression of X-linked inhibitor of apoptosis in human hepatocellular carcinoma. International journal of molecular medicine.

[26] Sakemi R, Yano H, Ogasawara S, Akiba J, Nakashima O, Fukahori S, Sata M, Kojiro M (2007). X-linked inhibitor of apoptosis (XIAP) and XIAP-associated factor-1 expressions and their relationship to apoptosis in human hepatocellular carcinoma and non-cancerous liver tissues. Oncology reports.

[27] Shi YH, Ding WX, Zhou J, He JY, Xu Y, Gambotto AA, Rabinowich H, Fan J, Yin XM (2008). Expression of X-linked inhibitor-of-apoptosis protein in hepatocellular carcinoma promotes metastasis and tumor recurrence. Hepatology.

[28] Kalra M, Mayes J, Assefa S, Kaul AK, Kaul R (2008). Role of sex steroid receptors in pathobiology of hepatocellular carcinoma. World journal of gastroenterology : WJG.

[29] Nilsson S, Makela S, Treuter E, Tujague M, Thomsen J, Andersson G, Enmark E, Pettersson K, Warner M, Gustafsson JA (2001). Mechanisms of estrogen action. Physiological reviews.

[30] Castellano L, Giamas G, Jacob J, Coombes RC, Lucchesi W, Thiruchelvam P, Barton G, Jiao LR, Wait R, Waxman J, Hannon GJ, Stebbing J (2009). The estrogen receptor-alpha-induced microRNA signature regulates itself and its transcriptional response. Proceedings of the National Academy of Sciences of the United States of America.

[31] Robertson CN, Roberson KM, Padilla GM, O'Brien ET, Cook JM, Kim CS, Fine RL (1996). Induction of apoptosis by diethylstilbestrol in hormone-insensitive prostate cancer cells. Journal of the National Cancer Institute.

[32] Qin J, Liu M, Ding Q, Ji X, Hao Y, Wu X, Xiong J (2014). The direct effect of estrogen on cell viability and apoptosis in human gastric cancer cells. Molecular and cellular biochemistry.

[33] Selbach M, Schwanhausser B, Thierfelder N, Fang Z, Khanin R, Rajewsky N (2008). Widespread changes in protein synthesis induced by microRNAs. Nature.

[34] Baek D, Villen J, Shin C, Camargo FD, Gygi SP, Bartel DP (2008). The impact of microRNAs on protein output. Nature.

[35] Zhang X, Liu S, Hu T, He Y, Sun S (2009). Up-regulated microRNA-143 transcribed by nuclear factor kappa B enhances hepatocarcinoma metastasis by repressing fibronectin expression. Hepatology.

[36] Yang X, Zhang XF, Lu X, Jia HL, Liang L, Dong QZ, Ye QH, Qin LX (2014). MicroRNA-26a suppresses angiogenesis in human hepatocellular carcinoma by targeting hepatocyte growth factor-cMet pathway. Hepatology.

[37] Takaki Y, Saito Y, Takasugi A, Toshimitsu K, Yamada S, Muramatsu T, Kimura M, Sugiyama K, Suzuki H, Arai E, Ojima H, Kanai Y, Saito H (2014). Silencing of microRNA-122 is an early event during hepatocarcinogenesis from non-alcoholic steatohepatitis. Cancer science.

[38] Bandiera S, Pfeffer S, Baumert TF, Zeisel MB (2014). miR-122 - A key factor and therapeutic target in liver disease. Journal of hepatology.

[39] Yang F, Yin Y, Wang F, Wang Y, Zhang L, Tang Y, Sun S (2010). miR-17-5p Promotes migration of human hepatocellular carcinoma cells through the p38 mitogen-activated protein kinase-heat shock protein 27 pathway. Hepatology.

[40] Huang S, He X, Ding J, Liang L, Zhao Y, Zhang Z, Yao X, Pan Z, Zhang P, Li J, Wan D, Gu J (2008). Upregulation of miR-23a approximately 27a approximately 24 decreases transforming growth factor-beta-induced tumor-suppressive activities in human hepatocellular carcinoma cells. International journal of cancer Journal international du cancer.

[41] Chen X, Ba Y, Ma L, Cai X, Yin Y, Wang K, Guo J, Zhang Y, Chen J, Guo X, Li Q, Li X, Wang W, Wang J, Jiang X, Xiang Y (2008). Characterization of microRNAs in serum: a novel class of biomarkers for diagnosis of cancer and other diseases. Cell research.

[42] Chhabra R, Adlakha YK, Hariharan M, Scaria V, Saini N (2009). Upregulation of miR-23a-27a-2-2 cluster induces caspase-dependent and -independent apoptosis in human embryonic kidney cells. PloS one.

[43] Yang X, Zhou Y, Peng S, Wu L, Lin HY, Wang S, Wang H (2012). Differentially expressed plasma microRNAs in premature ovarian failure patients and the potential regulatory function of mir-23a in granulosa cell apoptosis. Reproduction.

[44] Kent OA, Mendell JT (2006). A small piece in the cancer puzzle: microRNAs as tumor suppressors and oncogenes. Oncogene.

[45] Cohen A, Smith Y (2014). Estrogen regulation of microRNAs, target genes, and microRNA expression associated with vitellogenesis in the zebrafish. Zebrafish.

[46] Chang TC, Wentzel EA, Kent OA, Ramachandran K, Mullendore M, Lee KH, Feldmann G, Yamakuchi M, Ferlito M, Lowenstein CJ, Arking DE, Beer MA, Maitra A, Mendell JT (2007). Transactivation of miR-34a by p53 broadly influences gene expression and promotes apoptosis. Molecular cell.

[47] He L, He X, Lim LP, de Stanchina E, Xuan Z, Liang Y, Xue W, Zender L, Magnus J, Ridzon D, Jackson AL, Linsley PS, Chen C, Lowe SW, Cleary MA, Hannon GJ (2007). A microRNA component of the p53 tumour suppressor network. Nature.

[48] Cuddihy AR, Bristow RG (2004). The p53 protein family and radiation sensitivity: Yes or no?. Cancer metastasis reviews.

[49] Siegel C, Li J, Liu F, Benashski SE, McCullough LD (2011). miR-23a regulation of X-linked inhibitor of apoptosis (XIAP) contributes to sex differences in the response to cerebral ischemia. Proceedings of the National Academy of Sciences of the United States of America.

[50] Wang N, Zhu M, Wang X, Tan HY, Tsao SW, Feng Y (2014). Berberine-induced tumor suppressor p53 up-regulation gets involved in the regulatory network of MIR-23a in hepatocellular carcinoma. Biochimica et biophysica acta.

[51] Krek A, Grun D, Poy MN, Wolf R, Rosenberg L, Epstein EJ, MacMenamin P, da Piedade I, Gunsalus KC, Stoffel M, Rajewsky N (2005). Combinatorial microRNA target predictions. Nature genetics.

[52] Lewis BP, Burge CB, Bartel DP (2005). Conserved seed pairing, often flanked by adenosines, indicates that thousands of human genes are microRNA targets. Cell.

