# Supplementary material for: Estradiol induces apoptosis via activation of miRNA-23a and p53: implication for gender difference in liver cancer development
Source: Oncotarget. 2015 Sep 30;6(33):34941–52. doi: 10.18632/oncotarget.5472 (PMC4741500; doi:10.18632/oncotarget.5472)
Supplement: Supplementary file 1 [file oncotarget-06-34941-s001.pdf]

SUPPLEMENTARY TABLES AND FIGURES

Supplementary Table S1: Primers and oligonucleotides used in this study

Name	Sequence (5′ to 3′)
XIAP RT-F	GAAGACCCTTGGGAACAACA
XIAP RT-R	CGCCTTAGCTGCTCTTCAGT
p53 RT-F	GTTCCGAGAGCTGAATGAGG
p53 RT-R	TCTGAGTCAGGCCCTTCTGT
ERα RT-F	GACCAACCTGGCAGACAGGGAGC
ERα RT-R	AACCGAGATGATGTAGCCAGCAGC
Caspase-3 RT-F	AGAACTGGACTGTGGCATTGAG
Caspase-3 RT-R	GCTTGTCGGCATACTGTTTCAG
Caspase-7 RT-F	CTACCGCCGTGGGAACGATG
Caspase-7 RT-R	AGGCCCATACCTGTCACTTTATC
GAPDH RT-F	CAAATTCCATGGCACCGTCA
GAPDH RT-R	TCTCGCTCCTGGAAGATGGTGA
siRNA-ERα	GGAUUUGACCCUCCAUGAU
siRNA-p53	GGAAAUUUGCGUGUGGAGU

Supplementary Table S2: Raw data of miRNA PCR array analysis on the effects of estrogen (E2) treatment on alteration of 84 apoptotic miRNAs expression in SNU-387 cells (positive value indicated fold of upregulation of miRNAs and negative value indicated fold of downregulation of miRNAs).

Supplementary Table S3: List of genes related to apoptosis and predicted to be target genes of miR-23a

Gene	Symbol	NCBI reference	PicTar score	Targetscan Score
X-linked inhibitor of apoptosis	XIAP	NM_001167	#	–0.26
BCL2/adenovirus E1B 19kDa interacting protein 3-like	BNIP3L	NM_004331	0.65	–0.05
Caspase-7	CASP7	NM_001227	4.67	–0.18
Fas (TNF receptor superfamily, member 6)	FAS	NM_000043	#	–0.33
Topoisomerase (DNA) I	TOP1	NM_003286	4.02	–0.52
Phosphatase and tensin homolog	PTEN	NM_000314	#	–0.06
Interleukin 6 receptor	IL6R	NM_000565	4.48	–0.17
Signal transducer and activator of transcription 5B	STAT5B	NM_012448	3.22	–0.27
Suppressor of cytokine signaling 6	SOCS6	NM_004232	6.34	–0.29
Tumor necrosis factor, alpha-induced protein 3	TNFAIP6	NM-007115	1.96	–0.3
p21 protein (Cdc42/Rac)-activated kinase 6	PAK6	NM_020168	4.28	–0.16

Predictions were performed by PicTar (Krek et al., 2005) (http://pictar.bio.nyu.edu/) and TargetScan (Lewis et al., 2003) (http://www.targetscan.org/). Larger PicTar scores and more negative TargetScan scores indicate a higher likelihood. # No target predicted.

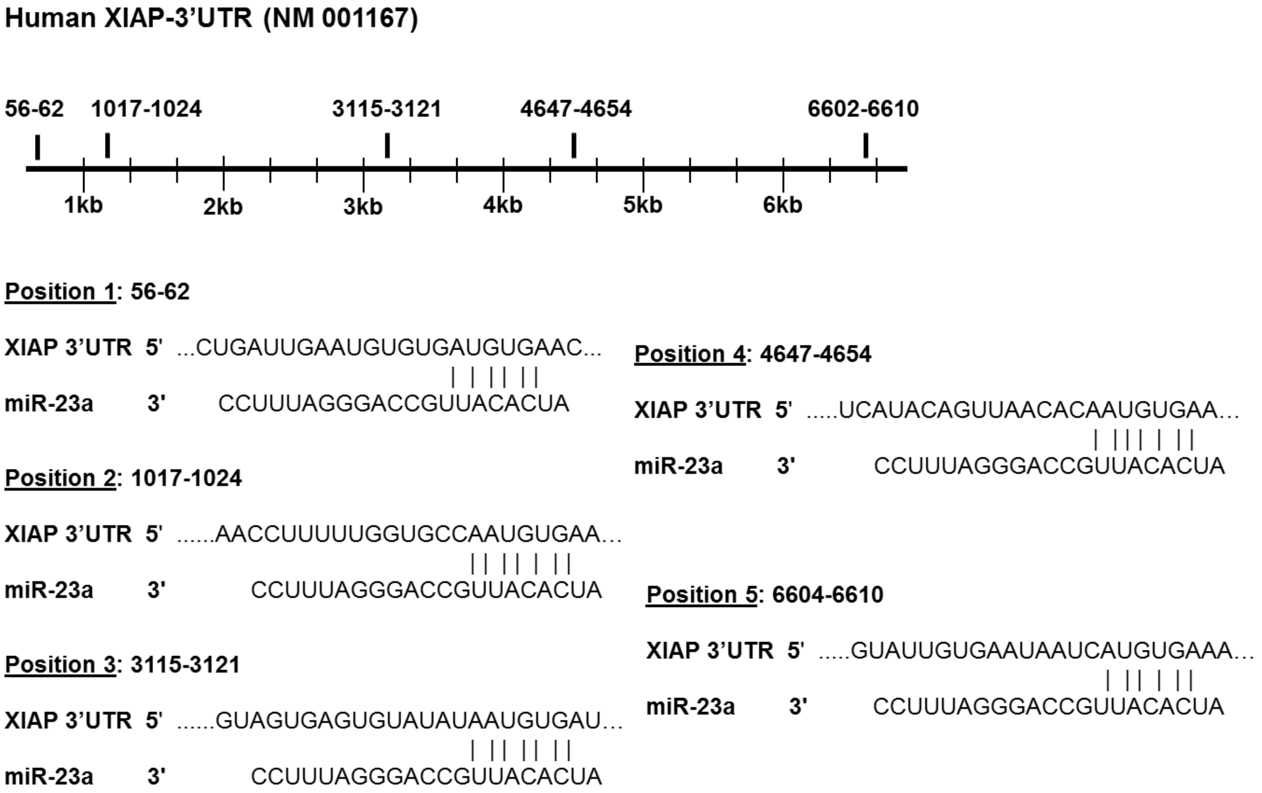

Supplementary Figure S1: The possible target sites in the XIAP 3′UTR interact with miR-23a were predicted by TargetScan software (conserved target sites: 1017–1024 and 3115–3121; unconserved target sites: 56–62, 4647–4654 and 6604–6610).

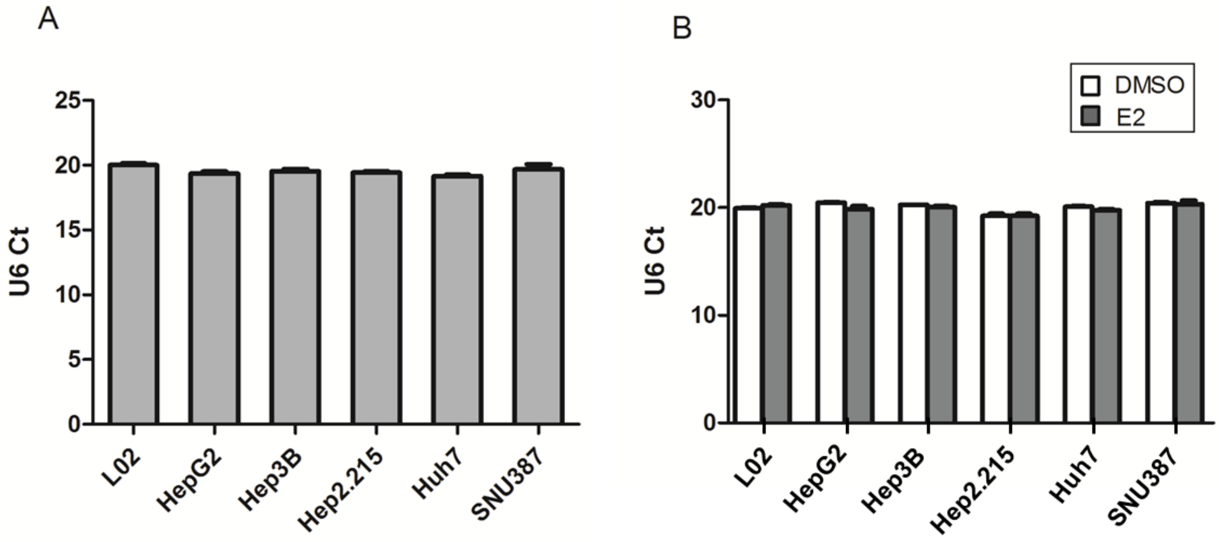

Supplementary Figure S2: Selection of U6 as the normalizer for relative quantitation of miRNA expression. A. Expression levels of U6 in six liver cell lines. **B.** Expression of U6 in the six liver cell lines was not response to E2 (10^{-8}M) and vehicle (DMSO) treatment. Values are given as real-time PCR cycle threshold numbers (Ct values).

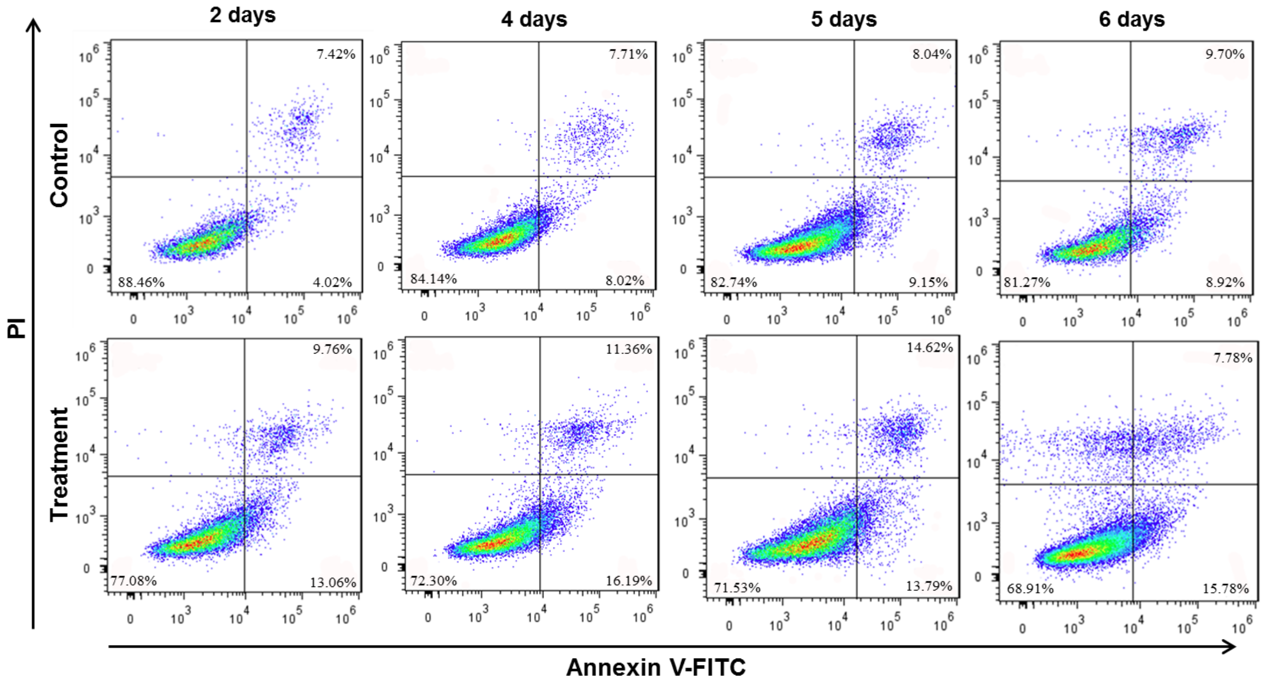

Supplementary Figure S3: Apoptotic effect of estrogen (E2) on SNU-387 cells. Induction of cell death was observed in SNU-387 cells treated with 10^{-8}M E2 for different times (2, 3, 5, 6 days). Cells were treated as mentioned above, stained with Annexin-V FITC and propidium iodide (PI), and then analyzed by flow cytometry. Representative results from three independent experiments are shown. Numbers indicated the percentage of cells in each quadrant by flow cytometry analysis.
