# Supplementary material for: Estradiol induces apoptosis via activation of miRNA-23a and p53: implication for gender difference in liver cancer development
Source: Oncotarget. 2015 Sep 30;6(33):34941–52. doi: 10.18632/oncotarget.5472 (PMC4741500; doi:10.18632/oncotarget.5472)
Supplement: Supplementary file 2 [file oncotarget-06-34941-s002.docx]

**Supplementary Table S2**

Raw data of miRNA PCR array analysis on the effects of estrogen (E2) treatment on alteration of 84 apoptotic miRNAs expression in SNU-387 cells (positive value indicated fold of upregulation of miRNAs and negative value indicated fold of downregulation of miRNAs).

| **PCR Array Catalog #: MIHS-114Z** | | **AVG Ct** | **AVG Ct** | **AVG Delta(Ct) (Ct(Gene of Interest) - Ave Ct(House Keeping Gene))** | | **2^(-Avg.(Delta(Ct))** | | **Fold Change (comparing to control group)** | **Up-Down Regulation (comparing to control group)** |
| --- | --- | --- | --- | --- | --- | --- | --- | --- | --- |
| **Position** | **Mature ID** | **Control Group** | **Treatment Group** | **Control Group** | **Treatment Group** | **Control Group** | **Treatment 1** | **Fold Change** | **Fold Regulation** |
| A01 | hsa-let-7a-5p | 20.52 | 22.83 | -2.591667 | -2.415 | 6.027947 | 5.333195 | 0.8847 | -1.1303 |
| A02 | hsa-let-7c-5p | 26.17 | 26.87 | 3.058333 | 1.625 | 0.120047 | 0.32421 | 2.7007 | 2.7007 |
| A03 | hsa-let-7e-5p | 23.07 | 24.28 | -0.041667 | -0.965 | 1.029302 | 1.952064 | 1.8965 | 1.8965 |
| A04 | hsa-let-7g-5p | 23.12 | 25.29 | 0.008333 | 0.045 | 0.99424 | 0.96929 | 0.9749 | -1.0257 |
| A05 | hsa-miR-1 | 33.92 | 34.58 | 10.808333 | 9.335 | 0.000558 | 0.001548 | 2.7766 | 2.7766 |
| A06 | hsa-miR-101-3p | 27.17 | 30.55 | 4.058333 | 5.305 | 0.060023 | 0.025295 | 0.4214 | -2.3729 |
| A07 | hsa-miR-106b-5p | 21.69 | 24.66 | -1.421667 | -0.585 | 2.678948 | 1.500039 | 0.5599 | -1.7859 |
| A08 | hsa-miR-122-5p | 33.41 | 34.33 | 10.298333 | 9.085 | 0.000794 | 0.001841 | 2.3187 | 2.3187 |
| A09 | hsa-miR-125a-5p | 20.68 | 21.71 | -2.431667 | -3.535 | 5.395163 | 11.591537 | 2.1485 | 2.1485 |
| A10 | hsa-miR-125b-5p | 21.19 | 23.05 | -1.921667 | -2.195 | 3.788605 | 4.578897 | 1.2086 | 1.2086 |
| A11 | hsa-miR-128-3p | 25.71 | 26.91 | 2.598333 | 1.665 | 0.165129 | 0.315344 | 1.9097 | 1.9097 |
| A12 | hsa-miR-1285-3p | 29.26 | 32.88 | 6.148333 | 7.635 | 0.014098 | 0.005031 | 0.3568 | -2.8024 |
| B01 | hsa-miR-133a-3p | 34.94 | 35 | 11.828333 | 9.755 | 0.000275 | 0.001157 | 4.2086 | 4.2086 |
| B02 | hsa-miR-133b | 35 | 35 | 11.888333 | 9.755 | 0.000264 | 0.001157 | 4.3873 | 4.3873 |
| B03 | hsa-miR-134-5p | 34.85 | 35 | 11.738333 | 9.755 | 0.000293 | 0.001157 | 3.9541 | 3.9541 |
| B04 | hsa-miR-141-3p | 35 | 35 | 11.888333 | 9.755 | 0.000264 | 0.001157 | 4.3873 | 4.3873 |
| B05 | hsa-miR-143-3p | 30.17 | 34.7 | 7.058333 | 9.455 | 0.007503 | 0.001425 | 0.1899 | -5.2659 |
| B06 | hsa-miR-144-3p | 35 | 35 | 11.888333 | 9.755 | 0.000264 | 0.001157 | 4.3873 | 4.3873 |
| B07 | hsa-miR-145-5p | 26.5 | 29.53 | 3.388333 | 4.285 | 0.095501 | 0.051296 | 0.5371 | -1.8618 |
| B08 | hsa-miR-146a-5p | 21.02 | 22.3 | -2.091667 | -2.945 | 4.262402 | 7.700756 | 1.8067 | 1.8067 |
| B09 | hsa-miR-149-3p | 26.83 | 30.43 | 3.718333 | 5.185 | 0.075975 | 0.027489 | 0.3618 | -2.7638 |
| B10 | hsa-miR-153-3p | 35 | 35 | 11.888333 | 9.755 | 0.000264 | 0.001157 | 4.3873 | 4.3873 |
| B11 | hsa-miR-15a-5p | 25.81 | 29.48 | 2.698333 | 4.235 | 0.154071 | 0.053105 | 0.3447 | -2.9012 |
| B12 | hsa-miR-15b-5p | 22.1 | 22.85 | -1.011667 | -2.395 | 2.016239 | 5.259771 | 2.6087 | 2.6087 |
| C01 | hsa-miR-16-5p | 21.04 | 24.16 | -2.071667 | -1.085 | 4.20372 | 2.121375 | 0.5046 | -1.9816 |
| C02 | hsa-miR-17-5p | 22.42 | 25.58 | -0.691667 | 0.335 | 1.615148 | 0.792784 | 0.4908 | -2.0373 |
| C03 | hsa-miR-181a-5p | 24.22 | 27.03 | 1.108333 | 1.785 | 0.46383 | 0.290176 | 0.6256 | -1.5984 |
| C04 | hsa-miR-181b-5p | 24.45 | 26.09 | 1.338333 | 0.845 | 0.395477 | 0.556711 | 1.4077 | 1.4077 |
| C05 | hsa-miR-181c-5p | 24.53 | 27.05 | 1.418333 | 1.805 | 0.374144 | 0.286181 | 0.7649 | -1.3074 |
| C06 | hsa-miR-181d-5p | 29.19 | 29.76 | 6.078333 | 4.515 | 0.014799 | 0.043737 | 2.9554 | 2.9554 |
| C07 | hsa-miR-183-5p | 34.19 | 33.7 | 11.078333 | 8.455 | 0.000462 | 0.00285 | 6.1617 | 6.1617 |
| C08 | hsa-miR-185-5p | 26.37 | 28.46 | 3.258333 | 3.215 | 0.104507 | 0.107693 | 1.0305 | 1.0305 |
| C09 | hsa-miR-186-3p | 34.54 | 35 | 11.428333 | 9.755 | 0.000363 | 0.001157 | 3.1895 | 3.1895 |
| C10 | hsa-miR-192-5p | 30.05 | 31.93 | 6.938333 | 6.685 | 0.008154 | 0.009719 | 1.192 | 1.192 |
| C11 | hsa-miR-193a-5p | 26.59 | 27.99 | 3.478333 | 2.745 | 0.089726 | 0.149167 | 1.6625 | 1.6625 |
| C12 | hsa-miR-193b-3p | 23.49 | 25.88 | 0.378333 | 0.635 | 0.769326 | 0.643941 | 0.837 | -1.1947 |
| D01 | hsa-miR-194-5p | 27.8 | 29.84 | 4.688333 | 4.595 | 0.038786 | 0.041378 | 1.0668 | 1.0668 |
| D02 | hsa-miR-195-5p | 21.95 | 24.79 | -1.161667 | -0.455 | 2.237157 | 1.370783 | 0.6127 | -1.632 |
| D03 | hsa-miR-200c-3p | 29.92 | 30.78 | 6.808333 | 5.535 | 0.008923 | 0.021567 | 2.4172 | 2.4172 |
| D04 | hsa-miR-203a | 35 | 35 | 11.888333 | 9.755 | 0.000264 | 0.001157 | 4.3873 | 4.3873 |
| D05 | hsa-miR-204-5p | 30.25 | 30.72 | 7.138333 | 5.475 | 0.007098 | 0.022483 | 3.1675 | 3.1675 |
| D06 | hsa-miR-205-5p | 35 | 35 | 11.888333 | 9.755 | 0.000264 | 0.001157 | 4.3873 | 4.3873 |
| D07 | hsa-miR-206 | 35 | 35 | 11.888333 | 9.755 | 0.000264 | 0.001157 | 4.3873 | 4.3873 |
| D08 | hsa-miR-20a-5p | 21.63 | 24.39 | -1.481667 | -0.855 | 2.792712 | 1.808759 | 0.6477 | -1.544 |
| D09 | hsa-miR-21-5p | 18.64 | 19.26 | -4.471667 | -5.985 | 22.187369 | 63.338026 | 2.8547 | 2.8547 |
| D10 | hsa-miR-210-3p | 25.96 | 28.61 | 2.848333 | 3.365 | 0.138857 | 0.097059 | 0.699 | -1.4306 |
| D11 | hsa-miR-212-3p | 32.49 | 35 | 9.378333 | 9.755 | 0.001503 | 0.001157 | 0.7702 | -1.2983 |
| D12 | hsa-miR-214-3p | 34.12 | 35 | 11.008333 | 9.755 | 0.000485 | 0.001157 | 2.3839 | 2.3839 |
| E01 | hsa-miR-218-5p | 28.91 | 29.6 | 5.798333 | 4.355 | 0.017969 | 0.048867 | 2.7195 | 2.7195 |
| E02 | hsa-miR-221-3p | 21.64 | 22.97 | -1.471667 | -2.275 | 2.773421 | 4.839976 | 1.7451 | 1.7451 |
| E03 | hsa-miR-222-3p | 21.65 | 23.69 | -1.461667 | -1.555 | 2.754264 | 2.938337 | 1.0668 | 1.0668 |
| E04 | hsa-miR-23a-3p | 19.93 | 20.86 | -3.181667 | -4.385 | 9.073547 | 20.893757 | 2.3027 | 2.3027 |
| E05 | hsa-miR-24-3p | 20.72 | 22.93 | -2.391667 | -2.315 | 5.247632 | 4.976047 | 0.9482 | -1.0546 |
| E06 | hsa-miR-25-3p | 23.37 | 24.5 | 0.258333 | -0.745 | 0.836053 | 1.675974 | 2.0046 | 2.0046 |
| E07 | hsa-miR-26a-5p | 21.12 | 22.67 | -1.991667 | -2.575 | 3.976962 | 5.95871 | 1.4983 | 1.4983 |
| E08 | hsa-miR-26b-5p | 23.17 | 23.36 | 0.058333 | -1.885 | 0.960373 | 3.693529 | 3.8459 | 3.8459 |
| E09 | hsa-miR-27a-3p | 21.34 | 24.58 | -1.771667 | -0.665 | 3.414482 | 1.585568 | 0.4644 | -2.1535 |
| E10 | hsa-miR-29a-3p | 20.65 | 23.59 | -2.461667 | -1.655 | 5.508527 | 3.149232 | 0.5717 | -1.7492 |
| E11 | hsa-miR-29b-3p | 27.36 | 29.93 | 4.248333 | 4.685 | 0.052617 | 0.038875 | 0.7388 | -1.3535 |
| E12 | hsa-miR-29c-3p | 21.05 | 24.15 | -2.061667 | -1.095 | 4.174683 | 2.136131 | 0.5117 | -1.9543 |
| F01 | hsa-miR-30a-5p | 22.43 | 25.61 | -0.681667 | 0.365 | 1.603992 | 0.776469 | 0.4841 | -2.0658 |
| F02 | hsa-miR-30b-5p | 24.1 | 26.72 | 0.988333 | 1.475 | 0.50406 | 0.359733 | 0.7137 | -1.4012 |
| F03 | hsa-miR-30c-5p | 22.65 | 24.29 | -0.461667 | -0.955 | 1.377132 | 1.93858 | 1.4077 | 1.4077 |
| F04 | hsa-miR-30d-5p | 24.7 | 26.93 | 1.588333 | 1.685 | 0.332555 | 0.311003 | 0.9352 | -1.0693 |
| F05 | hsa-miR-30e-5p | 22.27 | 25.67 | -0.841667 | 0.425 | 1.792119 | 0.744839 | 0.4156 | -2.4061 |
| F06 | hsa-miR-31-5p | 34.7 | 35 | 11.588333 | 9.755 | 0.000325 | 0.001157 | 3.5636 | 3.5636 |
| F07 | hsa-miR-32-5p | 31.08 | 35 | 7.968333 | 9.755 | 0.003993 | 0.001157 | 0.2898 | -3.4502 |
| F08 | hsa-miR-338-3p | 35 | 35 | 11.888333 | 9.755 | 0.000264 | 0.001157 | 4.3873 | 4.3873 |
| F09 | hsa-miR-34a-5p | 27.91 | 29.86 | 4.798333 | 4.615 | 0.035938 | 0.040808 | 1.1355 | 1.1355 |
| F10 | hsa-miR-34c-5p | 35 | 35 | 11.888333 | 9.755 | 0.000264 | 0.001157 | 4.3873 | 4.3873 |
| F11 | hsa-miR-365b-3p | 22.14 | 23.2 | -0.971667 | -2.045 | 1.961105 | 4.126733 | 2.1043 | 2.1043 |
| F12 | hsa-miR-378a-3p | 28.75 | 30.78 | 5.638333 | 5.535 | 0.020077 | 0.021567 | 1.0743 | 1.0743 |
| G01 | hsa-miR-409-3p | 32.29 | 33.87 | 9.178333 | 8.625 | 0.001726 | 0.002533 | 1.4675 | 1.4675 |
| G02 | hsa-miR-449a | 33.43 | 35 | 10.318333 | 9.755 | 0.000783 | 0.001157 | 1.4777 | 1.4777 |
| G03 | hsa-miR-451a | 33.81 | 35 | 10.698333 | 9.755 | 0.000602 | 0.001157 | 1.923 | 1.923 |
| G04 | hsa-miR-491-5p | 30.46 | 31.41 | 7.348333 | 6.165 | 0.006137 | 0.013936 | 2.271 | 2.271 |
| G05 | hsa-miR-497-5p | 31.14 | 33.67 | 8.028333 | 8.425 | 0.00383 | 0.00291 | 0.7596 | -1.3165 |
| G06 | hsa-miR-512-5p | 35 | 35 | 11.888333 | 9.755 | 0.000264 | 0.001157 | 4.3873 | 4.3873 |
| G07 | hsa-miR-542-3p | 35 | 35 | 11.888333 | 9.755 | 0.000264 | 0.001157 | 4.3873 | 4.3873 |
| G08 | hsa-miR-7-5p | 25.1 | 25.62 | 1.988333 | 0.375 | 0.25203 | 0.771105 | 3.0596 | 3.0596 |
| G09 | hsa-miR-708-5p | 32.64 | 33.79 | 9.528333 | 8.545 | 0.001354 | 0.002677 | 1.977 | 1.977 |
| G10 | hsa-miR-9-5p | 31.82 | 31.83 | 8.708333 | 6.585 | 0.002391 | 0.010416 | 4.357 | 4.357 |
| G11 | hsa-miR-92a-3p | 22.83 | 23.79 | -0.281667 | -1.455 | 1.215598 | 2.741566 | 2.2553 | 2.2553 |
| G12 | hsa-miR-98-5p | 26.42 | 27.17 | 3.308333 | 1.925 | 0.100947 | 0.26334 | 2.6087 | 2.6087 |
| H01 | cel-miR-39-3p | 35 | 35 | 11.888333 | 9.755 | 0.000264 | 0.001157 | 4.3873 | 4.3873 |
| H02 | cel-miR-39-3p | 35 | 35 | 11.888333 | 9.755 | 0.000264 | 0.001157 | 4.3873 | 4.3873 |
| H03 | SNORD61 | 23.57 | 26.92 | 0.458333 | 1.675 | 0.727827 | 0.313166 | 0.4303 | -2.3241 |
| H04 | SNORD68 | 23.71 | 24.93 | 0.598333 | -0.315 | 0.660517 | 1.244012 | 1.8834 | 1.8834 |
| H05 | SNORD72 | 24.81 | 27.82 | 1.698333 | 2.575 | 0.308142 | 0.167822 | 0.5446 | -1.8361 |
| H06 | SNORD95 | 23.32 | 24.57 | 0.208333 | -0.675 | 0.865537 | 1.596597 | 1.8446 | 1.8446 |
| H07 | SNORD96A | 23.39 | 25.16 | 0.278333 | -0.085 | 0.824543 | 1.060688 | 1.2864 | 1.2864 |
| H08 | RNU6-2 | 19.87 | 22.07 | -3.241667 | -3.175 | 9.458862 | 9.031715 | 0.9548 | -1.0473 |
| H09 | miRTC | 23.76 | 25.8 | 0.648333 | 0.555 | 0.638017 | 0.680657 | 1.0668 | 1.0668 |
| H10 | miRTC | 24.07 | 25.65 | 0.958333 | 0.405 | 0.514651 | 0.755236 | 1.4675 | 1.4675 |
| H11 | PPC | 19.99 | 20.09 | -3.121667 | -5.155 | 8.703928 | 35.629492 | 4.0935 | 4.0935 |
| H12 | PPC | 19.72 | 20.2 | -3.391667 | -5.045 | 10.495265 | 33.013862 | 3.1456 | 3.1456 |
